# *Streptococcus* co-opts a conformational lock in human plasminogen to facilitate streptokinase cleavage and bacterial virulence

**DOI:** 10.1074/jbc.RA120.016262

**Published:** 2020-11-24

**Authors:** Yetunde A. Ayinuola, Teresa Brito-Robinson, Olawole Ayinuola, Julia E. Beck, Diana Cruz-Topete, Shaun W. Lee, Victoria A. Ploplis, Francis J. Castellino

**Affiliations:** 1 W. M. Keck Center for Transgene Research, University of Notre Dame, Notre Dame, Indiana, USA; 2 Department of Chemistry and Biochemistry, University of Notre Dame, Notre Dame, Indiana, USA; 3 Department of Biological Sciences, University of Notre Dame, Notre Dame, Indiana, USA

**Keywords:** *S. pyogenes*, plasminogen, streptokinase, M-protein, bacterial virulence, protein interactions, conformational change, ligand binding, protein domains, AUC, analytical ultracentrifugation, EACA, ε-aminocaproic acid, GAS, gram-positive group A *Streptococcus pyogenes*, HC, heavy chain, hHC, human heavy chain, hLC, human light chain, hPg, human plasminogen, hPm, human plasmin, K2_hPg_, hPg kringle-2 module, LBS, lysine-binding site, LC, light chain, mPg, mouse Pg, mHC, mouse heavy chain, mLC, mouse light chain, MUGB, 4-methylumbelliferyl 4-guanidinobenzoate-HCl, PAM, plasminogen-binding group A streptococcal M-protein, Pm, plasmin, R, relaxed, SEC-MALS, size exclusion chromatography coupled with multiangle light scattering, SK, streptokinase, SPR, surface plasmon resonance, T, tight

## Abstract

Virulent strains of *Streptococcus pyogenes* (gram-positive group A *Streptococcus pyogenes* [GAS]) recruit host single-chain human plasminogen (hPg) to the cell surface—where in the case of Pattern D strains of GAS, hPg binds directly to the cells through a surface receptor, plasminogen-binding group A streptococcal M-protein (PAM). The coinherited Pattern D GAS-secreted streptokinase (SK2b) then accelerates cleavage of hPg at the R^561^-V^562^ peptide bond, resulting in the disulfide-linked two-chain protease, human plasmin (hPm). hPm localizes on the bacterial surface, assisting bacterial dissemination *via* proteolysis of host defense proteins. Studies using isolated domains from PAM and hPg revealed that the A-domain of PAM binds to the hPg kringle-2 module (K2_hPg_), but how this relates to the function of the full-length proteins is unclear. Herein, we use intact proteins to show that the lysine-binding site of K2_hPg_ is a major determinant of the activation-resistant T-conformation of hPg. The binding of PAM to the lysine-binding site of K2_hPg_ relaxes the conformation of hPg, leading to a greatly enhanced activation rate of hPg by SK2b. Domain swapping between hPg and mouse Pg emphasizes the importance of the Pg latent heavy chain (residues 1–561) in PAM binding and shows that while SK2b binds to both hPg and mouse Pg, the activation properties of streptokinase are strictly attributed to the serine protease domain (residues 562–791) of hPg. Overall, these data show that native hPg is locked in an activation-resistant conformation that is relaxed upon its direct binding to PAM, allowing hPm to form and provide GAS cells with a proteolytic surface.

Plasminogen (Pg) is a 791-amino acid single-chain plasma zymogen ([Glu^1^]-Pg), which upon proteolytic activation to the two-chain disulfide-linked serine protease, plasmin (Pm), *via* cleavage at the R^561^V^562^ peptide bond and additional loss of a N-terminal 77-residue activation peptide, catalyzes the degradation of fibrin clot. Besides this primary fibrinolytic function, Pm participates in several other pathophysiological events, often involving tissue remodeling and cell migration (1). One such process is the dissemination of bacteria during infections (2, 3), which is exemplified by gram-positive group A *Streptococcus pyogenes* (GAS) (4).

GAS expresses human plasminogen (hPg)/human plasmin (hPm) receptors, the most important of which is the surface-exposed cell wall–bound multicopy hPg-binding group A streptococcal M-protein (PAM) (4, 5, 6), that is used to serotype the >250 strains of GAS (7). PAM, present in a distinct class of M-Prt found only in skin-trophic Pattern D strains (8), enables GAS to directly bind host hPg with a strong affinity (K_D_ ∼1 nM) (3, 5). Activation of PAM-bound hPg by a specific coinherited secreted streptokinase (SK) subform, SK2b, furnishes the microorganism with a surface hPm activity that can potentially be employed to degrade barriers to its dissemination, *e.g.,* the protective fibrin surrounding the GAS within the human host (9, 10, 11, 12). Infections with GAS, particularly those strains that express the PAM subtype of the M-Prt, while mostly self-limiting and treatable, can be severe and degenerate into life-threatening diseases, *e.g.,* sepsis and necrotizing *fasciitis*, with an ∼40% mortality rate (13). Much of the virulence of Pattern D GAS is due to the specialized relationships between PAM, hPg, and SK2b. Unlike subforms of SK found in other GAS strains (*e.g.,* SK1 and SK2a), SK2b possesses very low activity for hPg activation in the absence of PAM, but the activation of hPg is greatly enhanced when hPg is bound to PAM (14, 15, 16, 17). Animal studies revealed that the virulence of PAM-expressing GAS strains is significantly attenuated upon inactivation of the PAM gene (4, 6), demonstrating that PAM is an important factor in the pathogenicity of GAS.

hPg is organized into seven structural domains, *viz,* a ∼77-residue amino-terminal activation peptide, followed by five consecutive ∼80-residue kringle domains (K1-K5), collectively designated the heavy chain (HC), downstream of which is an activation site and a carboxyl-terminal trypsin-like serine protease light chain (LC) domain (18). The kringle domains modulate hPg activation by binding to positive activation effector molecules, such as small-molecule lysine analogues, C-terminal protein lysine residues of proteins (19, 20), and peptidic side-chain lysine isosteres (21, 22). The amino acid residues that constitute the lysine-binding site (LBS) of hPg kringles have been well characterized by biochemical and high-resolution structural studies of isolated K1_hPg_ (23), K2_hPg_ (22, 24, 25), K4_hPg_ (26, 27), and K5_hPg_ (28, 29), in complex with ω-amino acid lysine analogues, such as ε-aminocaproic acid (EACA). For effective binding, LBSs require an anionic center*, viz.,* D^54^XD^56^ (using K1_hPg_ numbering from Cys^1^ of the isolated kringle) that interacts with the amino group of lysine analogues. The R^70^ cationic locus of the LBS interacts with COO^-^ of EACA, while Y^61^ and W^71^ form the hydrophobic groove that stabilizes the methylene groups of EACA (23). It has been shown that K1_hPg_ has the highest affinity for EACA, followed by K4_hPg_, K5_hPg_, and K2_hPg_ (K3_hPg_ does not possess a functional LBS) (28, 30). Using these same recombinant hPg kringle domains, along with truncated peptides from various PAM-type M-Prts, we demonstrated that the interaction between PAM and hPg is mediated by the LBS of K2_hPg_ along with one (a1) or two (a1, a2) lysine isostere(s) found in the NH_2_-terminal A-domain of PAM (21, 22, 31, 32, 33, 34). Although the LBS in K2_hPg_ displays the weakest affinity for lysine analogues (30, 35), PAM nonetheless exclusively tightly binds to this region of hPg. Since these kringle domains share extensive commonality in the LBS residues, it is concluded that binding exosites must exist in each kringle that modulates the affinity of hPg to PAM.

While mouse Pg (mPg) is highly homologous to hPg, and contains the same domain structure as hPg, mPg is well known not to be activated by SK and not to bind to PAM as efficiently as hPg (25). This perhaps explains the refractiveness of mPg to GAS infections unless applied to transgenic mice containing the hPg transgene (36). Since mice are the most commonly used surrogates for human diseases, we sought in the current study to expand the differences between hPg and mPg as virulence factors for GAS pathogenicity. Our approach involved the construction of chimeric variants of the two Pgs, as well as a single strategic mutation in K2_hPg_, to examine the strength of PAM binding and its effect on the activation rate of hPg. We correlated the biophysical properties of the Pg variants with their abilities to be activated to Pm through the virulence mechanism employed by Pattern D GAS. The results of this study are summarized in the current communication.

## Results

Since mice are heavily employed as models for human disease, we focused our work on differences at the protein levels that are responsible for the disparate pathogenicities between mice and humans as related to GAS infections.

In general, mice are poorly susceptible to infections by Pattern D GAS and thus are not optimal models for this disease. However, mice become very good models for these types of GAS infections when hPg DNA is transgenically inserted into the mouse genome, recapitulating many of the characteristics of human infections (36). A major reason for this is that mPg is not activated by GAS-secreted SK and mPg is not as tightly and effectively bound to the major Pg surface receptor, PAM (25). For these reasons, we have examined the basis of these differences between hPg and mPg through an approach that takes advantage of the well-known domainal nature of Pg, thus allowing the use of substitutions and exchanges of functional units of each Pg and assessment of the properties of the chimeric proteins.

### Sequence alignments of hPg and mPg/kringle domains of hPg

In order to examine the primary structural similarities and differences between hPg and mPg, their sequences were aligned (Fig. S1*A*). The alignments showed that the overall level of homology between hPg and mPg is ∼80%. Sequences within the latent HCs (shown in black) are ∼77% homologous between hPg and mPg, and sequences of the latent LCs (shown in blue) show ∼84% homology. Figure. S1*B* provides an alignment of the five kringle domains of hPg and shows that the lysine-binding residues (highlighted in red) of hPg are highly conserved across all the lysine-binding kringles (K1_hPg_, K2_hPg_, K4_hPg_, and K5_hPg_). K3_hPg_ does not bind to lysine because of a key mutation in its anionic center (DGD to DGK).

### Homogeneity, molecular weight, and Pg conformation

Parental Pgs (hPg and mPg), along with a set of chimeric Pgs, *viz.*, mouse heavy chain (mHC)-human light chain (hLC) and human heavy chain (hHC)-mouse light chain (mLC), and a point mutant, hPg[D^219^N], which eliminates by a single mutation the LBS in the K2 domain of hPg, were used in this work. Following expression of the Pgs in *Drosophila Schneider S2* cells, each variant was purified by lysine-Sepharose affinity chromatography (37). Typical protein yields ranged from 7 to 21 mg/l. The purities, molecular weights, and conformations of the Pg variants were examined by SDS-PAGE, size exclusion chromatography coupled with multiangle light scattering (SEC-MALS), and analytical ultracentrifugation (AUC).

**Figure 1:**
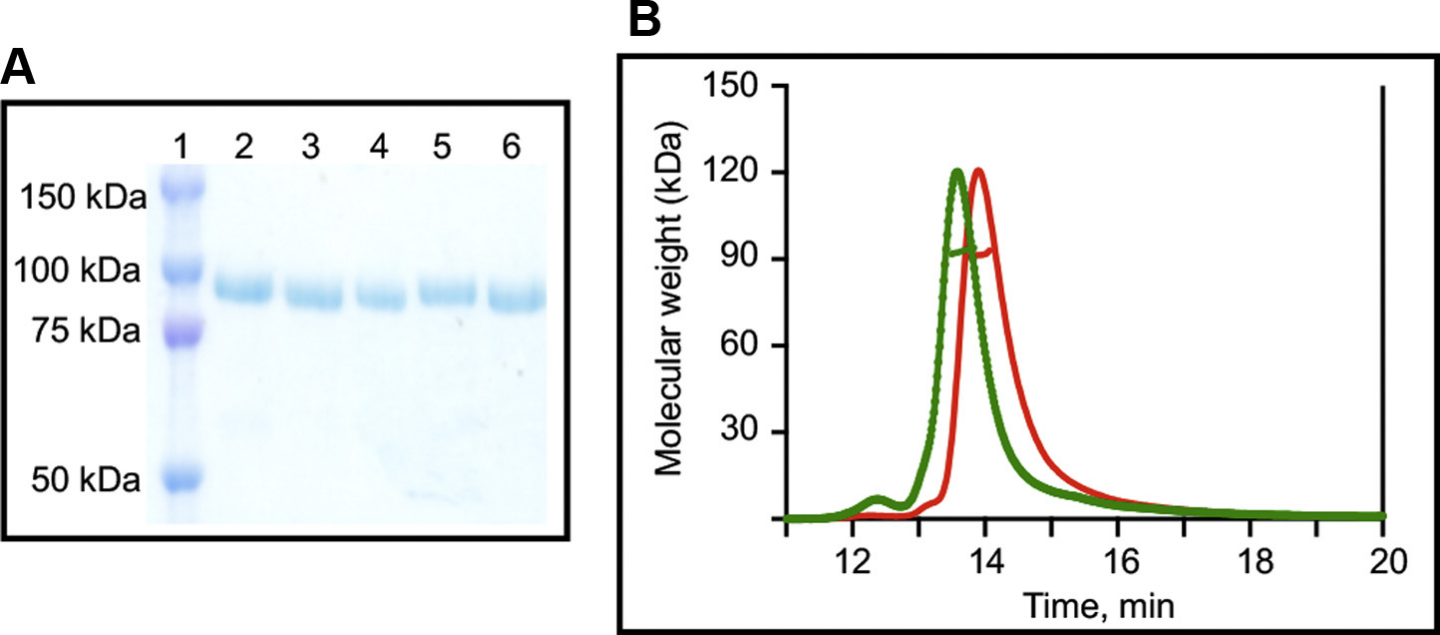
**Homogeneity test and molecular weight determinations**. (*A*) Electrophoretogram of Pg variants using 10% tris-glycine gels. Lanes marked 1 to 6 refer to the following: 1, protein marker; 2, hPg; 3, mPg, 4, mHC-hLC, 5, hHC-mLC, 6, hPg[D^219^N]. (*B*) Examples of SEC-MALS chromatograms of representative Pgs: red, hHC-mLC; green, mHC-hLC. One hundred microliters of each Pg was injected onto a Wyatt -030S5 SEC column (7.6 × 300 nm, 5 μm, 300 Å), equilibrated and eluted with PBS, pH 7.4, at a flow rate of 0.5 ml/min. The *horizontal thick lines* across each peak indicate the molecular weight of the Pg. SEC-MALS, size exclusion chromatography coupled to multiangle light scattering. hHC, human heavy chain; hLC, human light chain; mHC, mouse heavy chain; mLC, mouse light chain.

The gel electrophoretograms (Fig. 1*A*) show that all proteins were highly purified, with molecular weights close to their expected values. Some slight differences in mobility are observed, perhaps due to glycosylation differences. However, for precise determinations of the molecular weights of the chimeric Pgs, the purified proteins were first subjected to analysis by SEC-MALS, an example of which is shown in Figure 1*B*, as well as sedimentation equilibrium by AUC. The results, listed in Table 1, show agreement between the molecular weights using both analytical methods and the calculated molecular weights of each protein (minus the carbohydrate). These results demonstrate that highly purified full-length Pgs were obtained.

**Table 1:** Molecular weights of plasminogens

Plasminogen form	Molecular Weight, calculated kDa^a^	Molecular Weight, SEC-MALS kDa^b^	Molecular Weight., AUC kDa^c^
hPg	88.4	90.1 ± 0.9	90.7 ± 0.4
mPg	88.6	94.6 ± 0.3	92.5 ± 0.9
mHC-hLC	88.3	92.7 ± 0.7	91.9 ± 1.6
hHC-mLC	88.8	92.4 ± 0.7	90.8 ± 0.7
hPg[D^219^N]	88.4	95.3 ± 0.7	91.1 ± 1.5

a Molecular weight obtained from protein linear sequence calculated by ExPASy.

b Molecular weight determined by size exclusion chromatography coupled to multiangle light scattering (SEC-MALS).

c Molecular weight determined by analytical ultracentrifugation (AUC) at 20,000 rpm.

In the case of Pg, the sedimentation coefficient (S^0^_20,w_) value is highly diagnostic of the overall conformation and activatability of Pg. We have shown in numerous studies that fully intact Pgs from all species examined in Cl^-^-containing buffers provide a S_20,w_ value of ∼5.7 S, which represents the tight (T) conformation of Pg (19, 38, 39). In this state, the LBSs of the kringles are bound to side chains of other regions of the HC, including the activation peptide (40), thereby compacting the structure. The T form of hPg is not readily activatable. Relaxation of this conformation by addition of lysine and its analogues, *e.g.,* EACA, or removal of the 77-residue activation peptide, disrupts the side chain binding with the LBS of the kringle domains, thereby producing the highly activatable relaxed (R)-conformation with a S_20,w_ value that is reduced by ∼1 S (38, 39, 40, 41, 42, 43). The S_20,w_ values of each Pg (Table 2) show small differences in the T-conformations of the different chimeric proteins, perhaps reflective of altered interactions between their HCs and LCs, but the T-conformations of the HC were retained to a large degree in all proteins since addition of EACA reduced the S_20,w_ by ∼1.0 S. On the other hand, in the mutant hPg[D^219^N], the lowered S_20,w_ of 5.31 S partially disrupts the T-conformation of the hHC since a smaller additional change was found after addition of EACA. This observation has significant implications in its activation properties (*vide infra*).

**Table 2:** Sedimentation coefficients of plasminogens

Plasminogen form	S_20,w_ (S)^a^	S_20,w_ (S)^b^
hPg	5.70 ± 0.6	4.88 ± 0.17
mPg	5.68 ± 0.08	4.98 ± 0.01
mHC-hLC	5.49 ± 0.08	4.75 ± 0.12
hHC-mLC	5.43 ± 0.17	4.69 ± 0.05
hPg[D^219^N]	5.31 ± 0.04	4.78 ± 0.04

a 50 mM phosphate/100 mM NaCl, pH 7.4.

b 50 mM phosphate/100 mM NaCl/100 mM EACA, pH 7.4.

### The LC of hPg is responsible for the sensitivity to activation by SK2b

**Figure 2:**
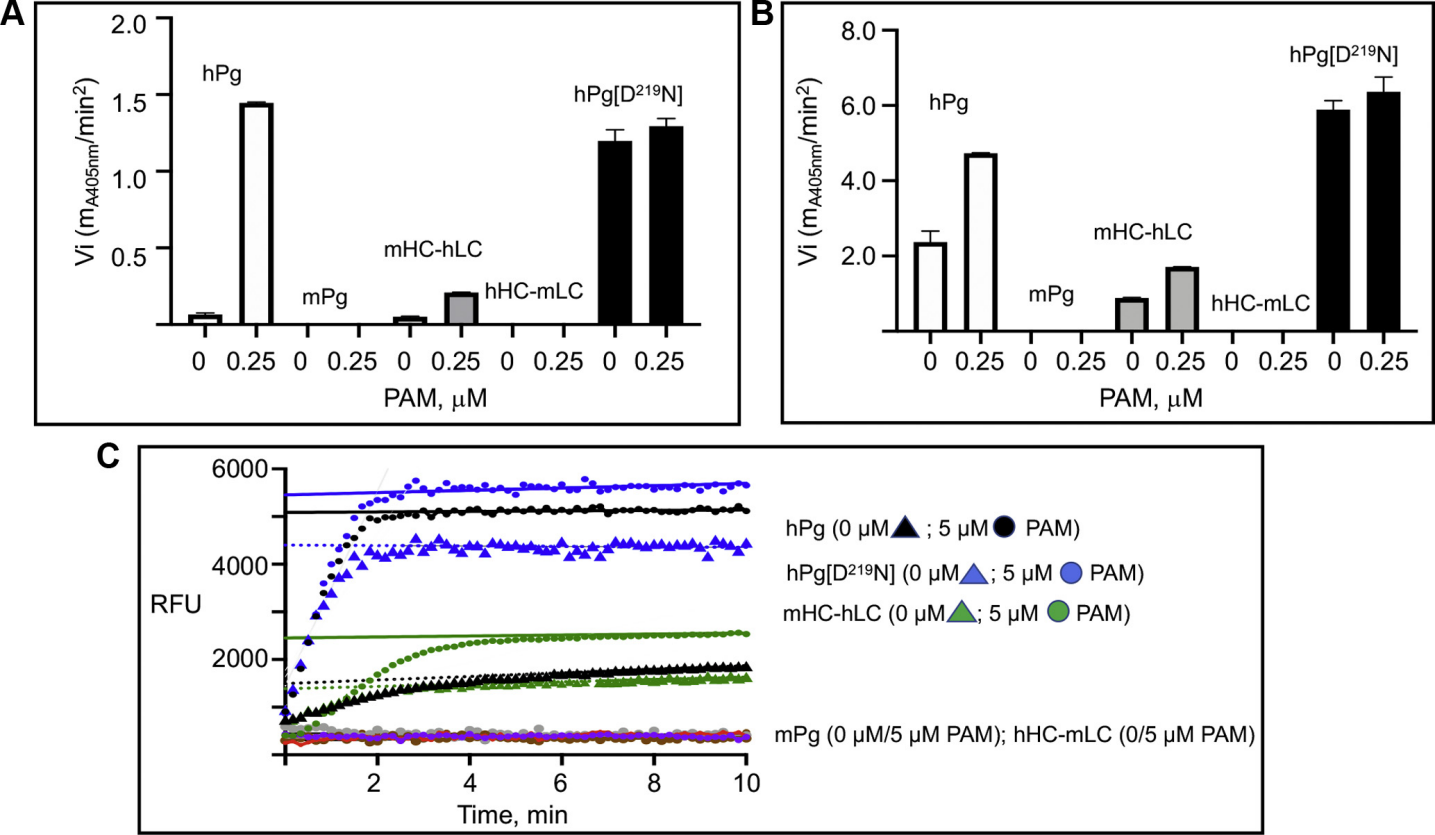
**Activation of Pg variants**. Assays were conducted in 10 mM Na^+^-Hepes/150 mM NaCl, pH 7.4, at 25 °C. The reaction mixtures contained 0.2 μM Pg, 0.25 mM S2251(H-D-Val-Leu-Lys-pNA), 0 or 250 nM PAM. The reaction was accelerated with (*A*) 5 nM SK2b; (*B*) 5 nM SK1. The release of p-nitroaniline was continuously monitored by A_405nm_ for 90 min. The initial velocities of each Pg activation were calculated from linear regions of plots of A_405nm_*vs* t^2^. (*C*) Generation of an active site in the 1:1 SK2b–Pg complexes. Pg (5 μM) was incubated with 0 or 5 μM PAM and 200 μM MUGB in 0.1 M phosphate/0.1 M NaCl pH 6.0. Complex formation was initiated by adding SK2b (10 μM). The relative fluorescence units (RFU) of methylumbelliferone generated upon SK2b–Pg complex formation, accompanied by cleavage of MUGB, was measured by excitation at 323 nm and emission at 445 nm. The extrapolation of the steady-state curve to zero at 0 (*dashed line*) and 5 μM PAM (*thick line*) indicates the pre–steady-state concentration of methylumbelliferone. MUGB, 4-methylumbelliferyl 4-guanidinobenzoate-HCl; PAM, plasminogen-binding group A streptococcal M-protein.

In studying the activation properties of the Pgs, the generation of Pm was coupled in a single reaction to hydrolysis of the chromogenic substrate, S2251, and the continual release of p-nitroaniline was monitored by A_405nm_ to provide an initial rate of activation (44). Figure 2*A* shows the activation rate profiles by SK2b for the parental Pgs (hPg and mPg), chimeric Pgs, and the point mutant hPg[D^219^N]. Consistent with our previous report (15), the rate of activation of hPg by SK2b is extremely slow in Cl^-^ buffers in the absence of PAM. A notable exception is found for hPg[D^219^N], in which a key residue of the LBS of K2_hPg_ was inactivated by mutagenesis, and led to a greatly enhanced activation rate by SK2b in the absence of PAM.

The rate of hPg activation by SK2b was highly stimulated by PAM (Fig. 2*A*). No form of Pg containing the mLC showed any propensity for activation by SK2b, with or without PAM. The stimulatory effect of PAM was observed on the activation of chimeric mHC-hLC, but was somewhat slower than that of hPg, possibly due to the two critical differences in residues 220 and 222 between the hHC and mHC (25). A single mutation in hPg, *viz.,* [D^219^N], abolished the stimulatory effect of PAM, as reflected by the greatly enhanced activation rate of this variant in the absence of PAM, which reached the same level as that of hPg/PAM and hPg[D^219^N]/PAM (Fig. 2*A*).

Because there is a coinheritance between SK2b and PAM that enables effective activation of hPg in strains of GAS that secrete SK2b (15), it is relevant to further understand the reason for the slow rate of activation of mHC-hLC as it relates to SK and PAM. Therefore, we investigated Pg activation by SK1, a class of SK not present in Pattern D GAS, that effectively activates hPg regardless of the presence of PAM (15, 45). Figure 2*B* clearly shows that the rates of activation of hPg, mHC-hLC, and hPg[D^219^N] by SK1 in the absence of PAM were faster than those of PAM-stimulated activations with SK2b. Rate enhancements by PAM were also observed for hPg, and mHC-hLC activations, but were understandably smaller than those of SK2b. Nevertheless, mHC-hLC, similar to the data obtained for the SK2b activation, could not achieve rates comparable to that of hPg and hPg[D^219^N] under the same conditions.

To more fully probe the reasons for the lack of activation of mPg by SK, we investigated the ability of mPg to form the initial active site in the SK–Pg complex that is essential to further activation of Pg by SK. Here, we determined that the single turnover fluorometric substrate, 4-methylumbelliferyl 4-guanidinobenzoate-HCl (MUGB), is cleaved by a stoichiometric complex SK2b/hPg, SK2b/mHC-hLC, and SK2b/hPg[D^219^N] to 19%, 18%, and 66%, respectively, in the absence of PAM, and to 78%, 33%, and 86%, respectively, in the presence of saturating [PAM]. SK2b/mPg and SK2b/hHC-mLC did not form an active site with or without PAM (Fig. 2*C*). This lack of initial active site formation within the mLC prevented further activation of Pg.

### LBS residue D^219^ of hPg is critical for PAM binding

**Figure 3:**
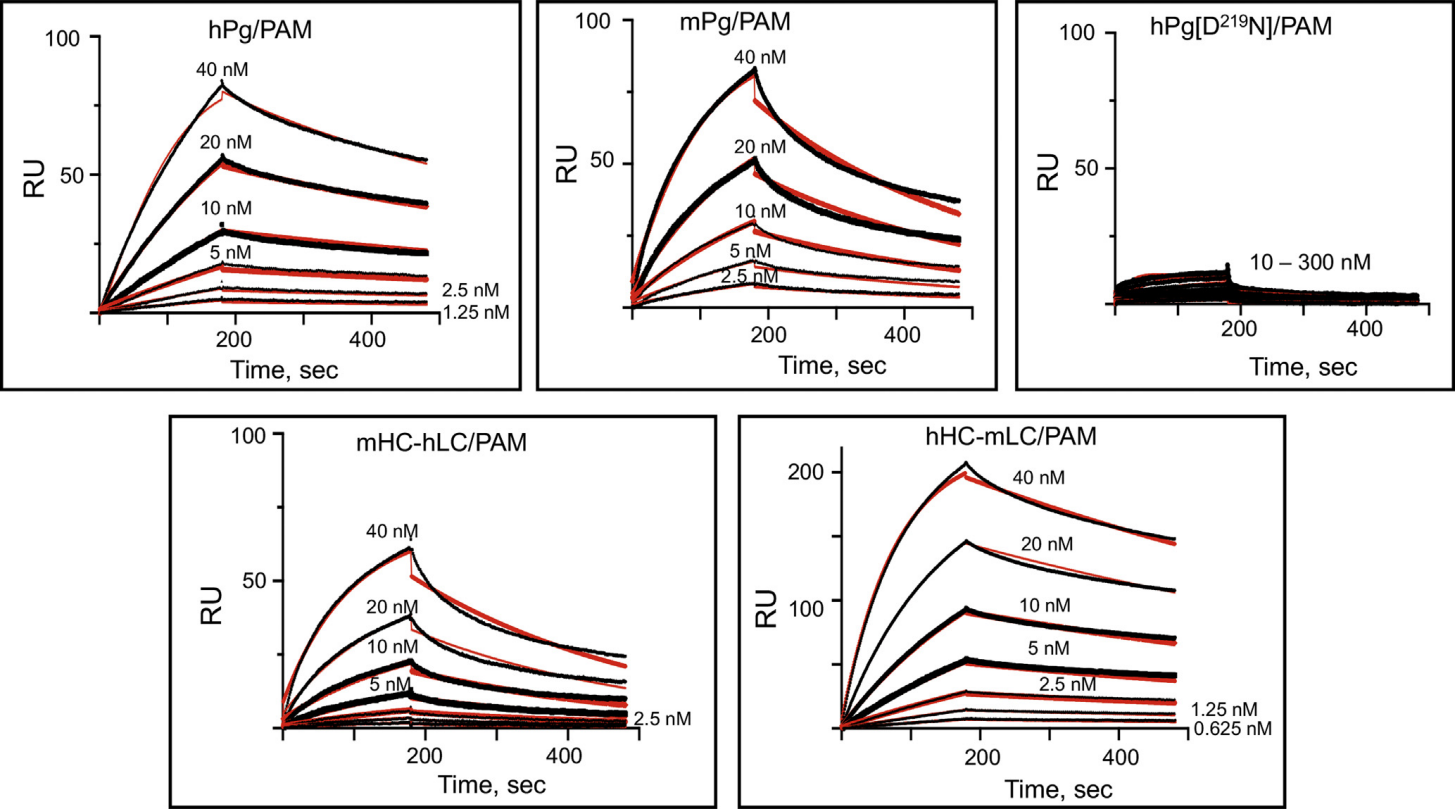
**SPR-based binding of Pg variants to PAM at 25 °C**. PAM was immobilized on a CM-5 chip. The concentrations of Pg variants used in the titrations are indicated in each plot. Sensorgrams representing the experimental data are shown in *black lines*, and best-fit curves are shown in *red lines*. The data were fit using a 1:1 Langmuir model. PAM, plasminogen-binding group A streptococcal M-protein; SPR, surface plasmon resonance.

A key residue of the LBS of the K2 domain, D^219^, was mutated to determine whether PAM binding was also ablated. If so, this would provide evidence that only the LBS of K2 was a determining factor in PAM binding to intact hPg. Figure 3 illustrates the surface plasmon resonance (SPR) sensorgrams generated for the interactions between the various Pgs and PAM. The kinetic parameters are summarized in Table 3. All Pg variants had K_D_ values for PAM in the low nM range, indicating high-affinity interactions with PAM. The K_D_ value of mPg is 14 nM, a value ∼10-fold higher than that of hPg. These differences in K_D_ values are reflections of the ∼5.5-fold faster off-rate (k_off_), of mPg upon binding to PAM, as compared to that of hPg. A swap in the LC between mPg and hPg did not decrease the k_off_ of mHC-hLC, as this variant had similar kinetic parameters to mPg. On the other hand, the hHC-mLC chimera binds PAM as tightly as hPg (K_D_ ∼3 nM). The binding affinities of both mHC-hLC and hHC-mLC chimeras, as compared to their parental Pgs, indicate that the LC plays a minimal role in the interaction of Pg with PAM. The important mutation present in the variant hPg[D^219^N] completely ablated the PAM binding site, demonstrating that PAM binding to hPg is primarily dependent on an intact LBS of K2_hPg_.

**Table 3:** Kinetics of binding of PAM to plasminogens at 25 °C

Plasminogen form	k_on_ (1/Msec × 10^4^)	k_off_ (1/sec × 10^4^)	Kd (nM)^a^
hPg	27 ± 1.1	6.0 ± 0.02	2.2 ± 0.1
mPg	26 ± 5.6	33 ± 1.0	14 ± 3.7
mHC-hLC	24 ± 1.3	38 ± 0.5	16 ± 1.1
hHC-mLC	39 ± 0.6	12 ± 0.5	3 ± 0.2
hPg[D^219^N]	nd^b^	nd	nd

a Kd values were calculated from the average values of k_off_/k_on_.

b Not detected.

### Binding of Pg to SK

**Figure 4:**
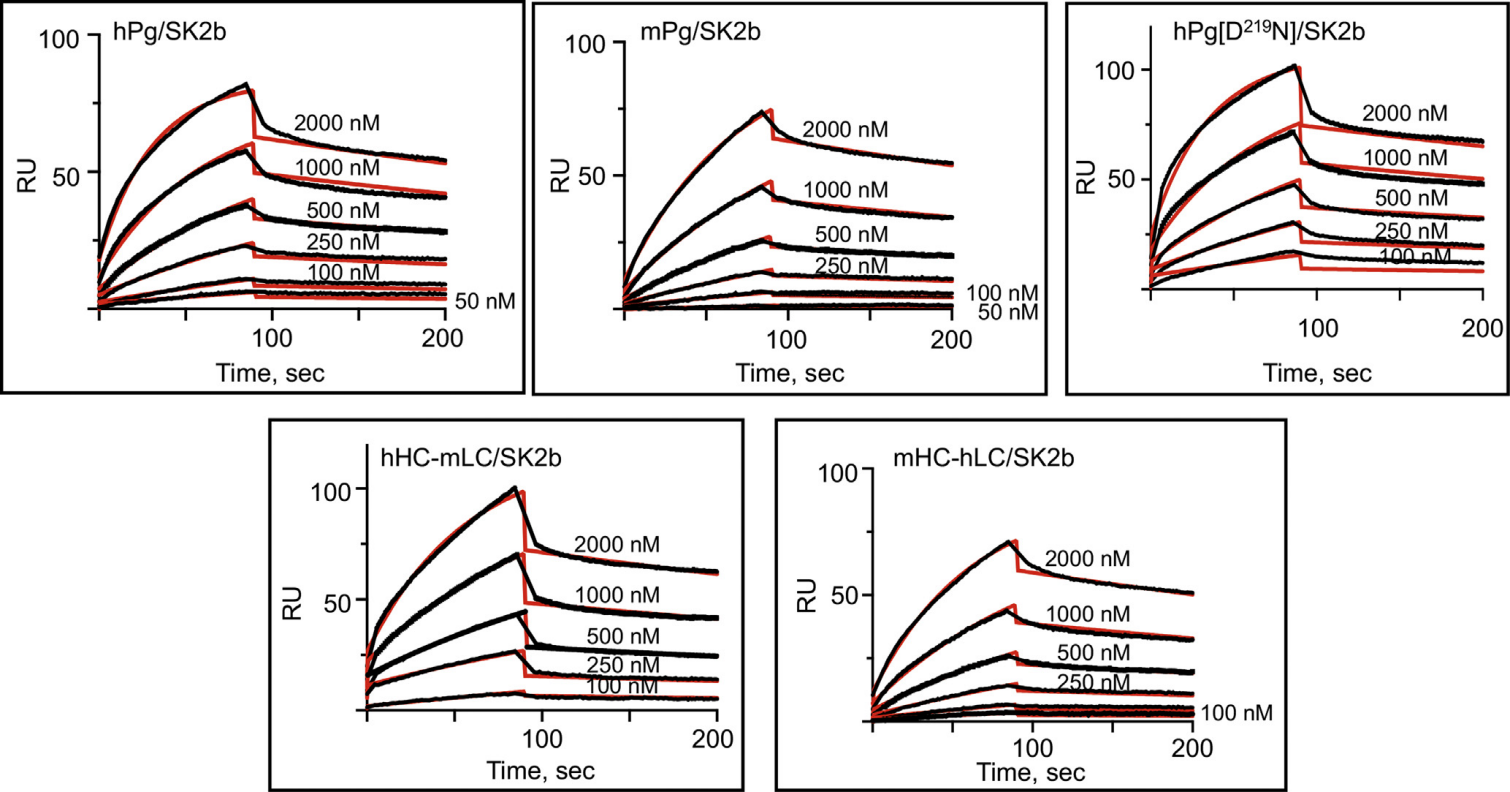
**SPR-based binding of SK2b to Pg variants at 25 °C**. Each Pg variant was immobilized on a CM-5 chip. The concentrations of SK are indicated in each plot. The sensorgrams represent the binding of SK2b to the immobilized Pg. The experimental data are shown in *black lines*, and best-fit curves are shown in *red lines*. The data were fit using 1:1 Langmuir model. SPR, surface plasmon resonance.

**Figure 5:**
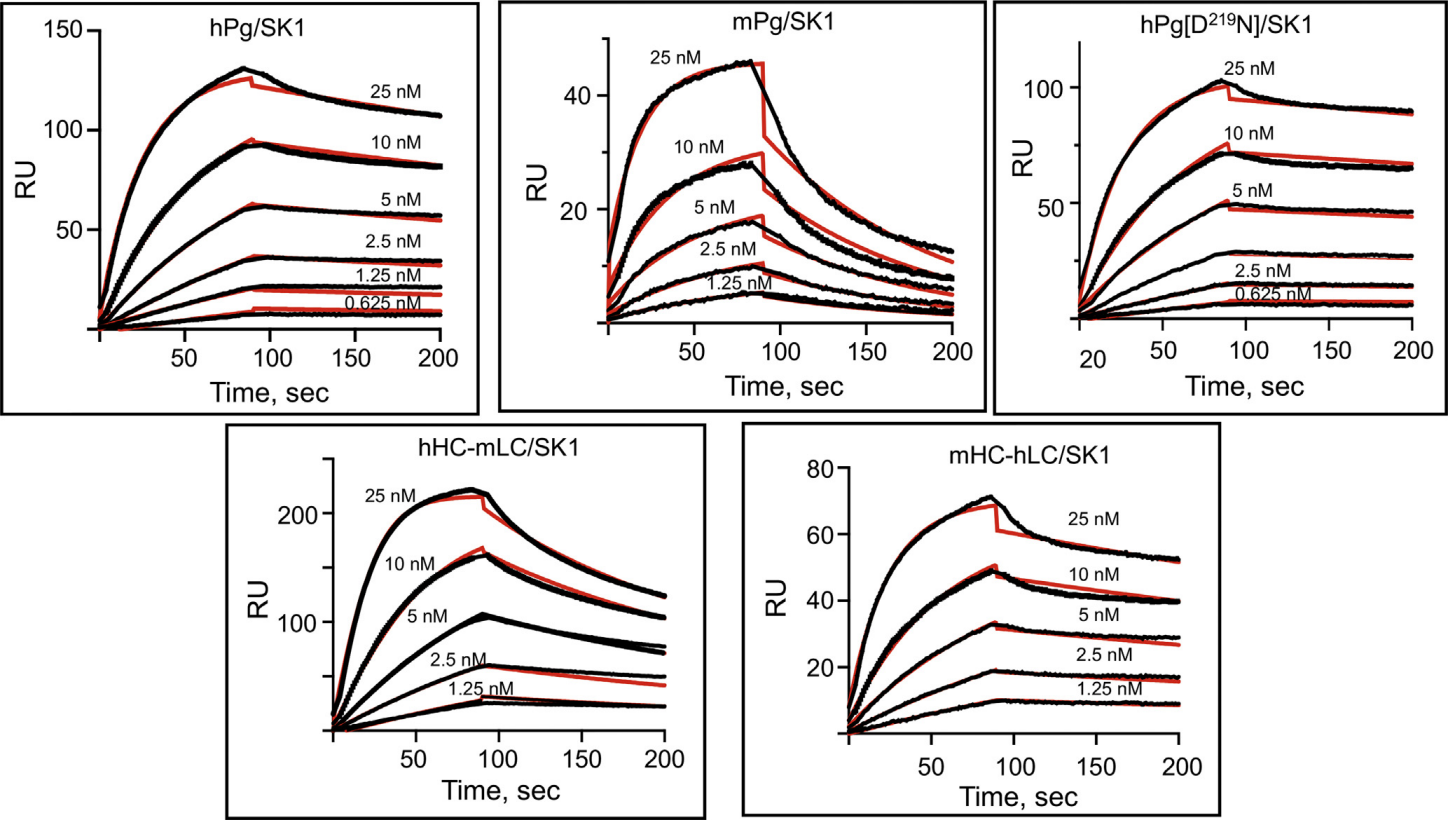
**SPR-based binding of SK1 to Pg variants at 25 °C**. Each Pg variant was immobilized on a CM-5 chip. The concentrations of SK are indicated in each plot. Sensorgrams representing the binding of SK1. The experimental data are shown in *black lines*, and best-fit curves are shown in *red lines*. The data were fit using 1:1 Langmuir model. SPR, surface plasmon resonance.

SPR experiments were also carried out to determine the influence of the HC on the binding of SK to the LC of hPg. We have shown in a previous study that the K_D_ of the hPg[S^741^A] (hPg in which the active site serine was replaced with alanine to prevent the conversion of hPg to hPm)–SK2b interaction is ∼two orders of magnitude weaker than that of SK1, as reflected in their K_D_ values (15). In the current study, the binding data of hPg determined in the presence of the rapid single turnover serine colorimetric protease inhibitor, p-nitrophenyl-p’guanidinobenzoate which functions similarly to the fluorimetric substrate, MUGB, and also prevents the conversion of hPg to hPm within the SK complex, thus halting further activation, agree with our initial findings of an ∼100-fold higher K_D_ due to a slower binding *k*_*on*_ of SK2b (Fig. 4, Table 4) than SK1 (Fig. 5, Table 5). This difference is also exhibited by the chimeric Pgs, all of which interacted with the both forms of SK. For SK1, the dissociation constants of all the Pg variants are in the very low nanomolar range (1–6 nM). A distinction, however, exists between the binding rate constants of Pgs with the hLC versus the mLC. SK1 dissociates ∼10x faster upon binding to mPg than its binding to hPg. This faster *k*_*off*_ appeared to be structurally compensated for in hHC-mLC, suggesting that stable binding between SK1 and Pg is determined by the LC and may be somewhat modulated by the Pg conformation induced by the HC. This *k*_*off*_ difference was not observed for the SK2b–Pg complexes. The difference between SK2b and SK1 is likely due to amino acid sequence variations in their β-domains, which have been shown earlier to be responsible for the higher *k*_*on*_ with corresponding high-affinity binding between SK1 and hPg (17). Overall, the binding data for the chimeric Pg variants with SK2b and SK1 show that, beyond slow association or rapid dissociation rates, the lack of activation of mPg and hHC-mLC, along with the delayed activation of mHC-hLC, are affected by properties other than simple direct binding. Most likely, the conformational rearrangement needed to form the initial active site does not occur with the mLC.

**Table 4:** Kinetics of binding of SK2b to plasminogens at 25 °C

Plasminogen form	k_on_ (1/Msec × 10^4^)	k_off_ (1/sec × 10^4^)	Kd (nM)^a^
hPg	1.8 ± 0.34	15 ± 0.02	85 ± 10
mPg	0.6 ± 0.6	16 ± 1.0	240 ± 20
mHC-hLC	0.8 ± 0.07	16 ± 0.5	202 ± 16
hHC-mLC	0.8 ± 0.6	14 ± 0.5	187 ± 6
hPg[D^219^N]	1.7 ± 0.22	12 ± 0.8	73 ± 14

a Kd values were calculated from the average values of k_off_/k_on_.

**Table 5:** Kinetics of binding of SK1 to plasminogens at 25 °C

Plasminogen form	k_on_ (1/Msec × 10^4^)	k_off_ (1/sec × 10^4^)	Kd (nM)^a^
hPg	165 ± 12	15 ± 3.3	0.9 ± 0.1
mPg	165 ± 5	102 ± 2.3	6.1 ± 0.3
mHC-hLC	157 ± 5	16 ± 0.4	1.0 ± 0.3
hHC-mLC	303 ± 14	59 ± 3.7	1.9 ± 0.1
hPg[D^219^N]	148 ± 15	5 ± 1	0.4 ± 0.1

a Kd values were calculated from the average values of k_off_/k_on_.

## Discussion

**Figure 6:**
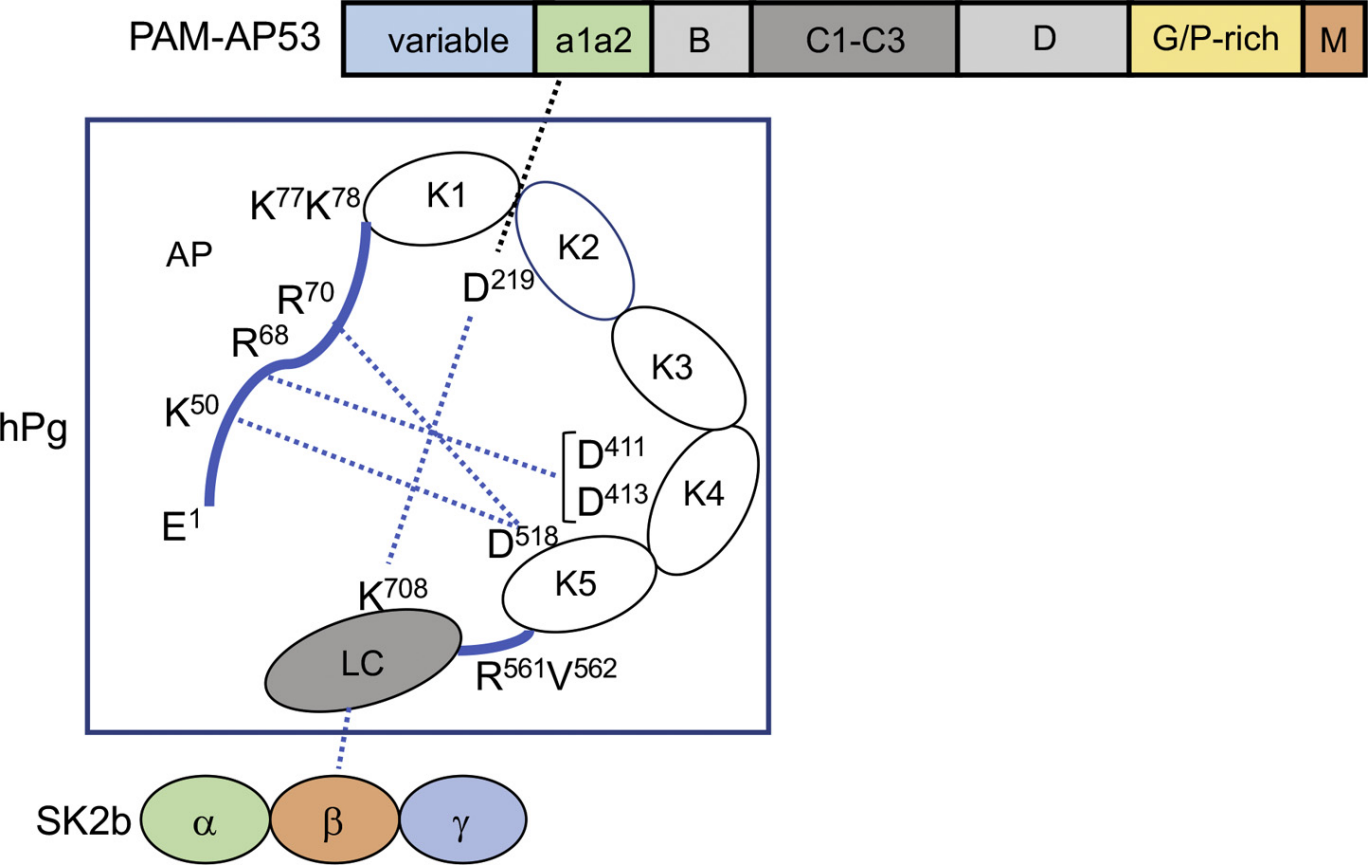
**Model of the lysine-binding site (LBS) interactions that are relevant to the T- and R-conformations of hPg and the stimulation of the SK2b-mediated activation of hPg by PAM**. Illustrated are the domain regions of PAM (top), hPg (middle), and SK2b (bottom). hPg is shown in its activation-resistant T-conformation, which is stabilized by side chain interactions of K^50^, R^68^, and R^70^ of the activation peptide (AP) with LBS residues in D^411^/D^413^ of hPg kringle 4 (K4) and D^518^ of hPg K5. The stability of the T-conformation is also afforded by the interaction of the critical (D^219^) residue of the LBS of hPg K2 with K^708^ of the serine protease (LC) domain of hPg. PAM interacts with the LBS of the K2 module of the T-conformation of hPg *via* lysine isosteric side chain residues in the a1a2 region of the PAM A domain. This interaction disrupts the D^219^-K^708^ bond and partially relaxes the T-conformation and exposes the R^561^-Val^562^ activation bond, thereby enhancing the activation rate of hPg. As hPm is formed, the K^77^-K^78^ peptide bond is cleaved, leading to the full R-conformation, which is highly activatable by any hPg activator. SK2b is initially bound to the hPg through residues in the β-domain of SK and the SP (light chain) region of hPg or hPm. LC, light chain; PAM, plasminogen-binding group A streptococcal M-protein; SK, streptokinase; SP, serine protease.

Intramolecular interactions that stabilize the activation-resistant T-conformation of hPg occur primarily between its 77-amino acid residue N-terminal activation peptide and the LBS of two of its five kringle domains (46), thereby explaining the conversion of the T to the R-conformation when the 77-residue activation peptide is removed and when ω-amino acids are added to displace the intramolecular interactions (42). Solving the X-ray crystal structure of hPg (46) led to a model (Fig. 6) in which K^50^ of the activation peptide forms a salt bridge with D^518^ of the LBS of K5_hPg_, R^68^ is appropriately distanced to interact with both D residues of the D^411^XD LBS of K4_hPg_, and R^70^ of the activation peptide is positioned to also coordinate with D^413^ of the LBS of K4_hPg_. The LC of hPg can also participate in maintaining the T-conformation through the formation of a salt bridge between the ε-NH_2_ of Lys^708^ and D^219^GE of the LBS of K2_hPg_. The cationic binding region of the LBS of K2_hPg,_ *viz.,* R^234^, forms a salt bridge with E^706^ of the hPg-LC. Also, the activation inhibitor, Cl^-^, can coordinate to R^234^ and W^235^ of the LBS of K_2hPg_ and the backbone nitrogen of K^708^ of the LC, thus expanding the interactions of K2_hPg_ with the LC. Therefore, elimination of the LBS of K2_hPg_ relaxed the T-conformation in such a way as to provide exposure of the R^561^-V^562^ activation cleavage site, leading to enhanced activation rates. Of all of the LBS-containing kringles, only the LBS of K1 does not appear to participate in intramolecular interactions to maintain the T-conformation. These observations support the conclusion that the LBS of K2_hPg_ is critical for maintenance of its T-conformation and elimination of this LBS, even by a simple mutation of hPg[D^219^N], allows the activator more ready access to the R^561^V^562^ cleavage site to SK2b/hPg’ and SK2b/hPm activator complexes.

In concert with studies using isolated kringle domains (21, 22, 24, 33, 47, 48), we demonstrated in the current study that the LBS of K2_hPg_ is the only kringle unit indispensable for interaction of hPg with PAM in the intact proteins. Examination of the effect of PAM on the SK2b-mediated activation of chimeric Pgs showed that contrary to the high activation rate enhancement by PAM observed for hPg, smaller differential rate increments exist between the unstimulated and the PAM-stimulated activation of mHC-hLC. The same trend was observed when a preformed active complex of SK2b-hPg/hPm was used as an activator, which clearly demonstrated that Pgs possessing the mHC respond less vigorously to stimulation by PAM even though they tightly bind PAM (data not shown). The highest K_D_ value for the binding of PAM to chimeric Pgs is ∼20 nM. Since PAM was utilized at a near-saturating concentration of 250 nM and hPg at 200 nM, it is expected that >90% of all Pgs should be in their PAM-bound forms. Hence, the lower stimulatory effect of PAM on mHC-hLC activation might be that the binding of PAM to the mHC does not promote/stabilize the most suitable conformation required by SK2b. This is suggested by the lack of formation of the initial required active site in the mPg–SK2b complex, thereby halting activation of mPg to mPm by SK.

The activation properties of the chimeric Pgs in reactions with SK1 and SK2b clearly demonstrate that the lack of activation of mPg by SK could be accounted for by the differences between its LC (mLC) and that of hPg (hLC). Other studies have shown that microplasminogen (μhPg; K^530^-N^791^), containing only the activation cleavage site (R^561^-Val^562^) and hLC domains, can bind to SK (49, 50), albeit with lower affinity than hPg, and form a catalytically active complex (51). Since μhPg is essentially the LC of hPg, these findings imply that the LC of Pg mediates binding to SK. The importance of the hLC in the formation of SK–hPg complex was further demonstrated in a study that reported the crystal structure of SK in complex with μhPm (52). Here, it was shown that several residues, including R^719^, which are close to H^603^ and D^646^ of the serine protease catalytic triad (H^603^/D^646^/S^741^), as well as residues 622 to 628 and residues 692 to 695 (autolysis loop), participate in hydrophobic clustering, hydrogen bond formation, electrostatic interactions, and van der Waals interactions with residues from SK. An overlay of the crystal structure of the μhPg–SK complex onto the hPg crystal structure shows that SK avoids interactions with the HC of hPg (53). However, that may not be the case since the HC of hPg was not available in μhPg. Thus, while supportive of other work, as revealed by the binding data in the current study, this conclusion must be tempered at this time.

While most of these residues are conserved between mPg and hPg, residues 624 to 628 are dissimilar. The divergence in residues 624 to 628 was also noted to be the case with bovine Pg (52), another SK-insensitive Pg. From our current binding data, the difference in the binding properties of hLC and mLC is minimal, suggesting that residues required for hPg and mPg binding to SK are conserved. Thus, complexes of mPg and hHC-mLC with SK must exist in their different activation mixtures. However, the lack of activation with this last set of Pgs rests in the inability of their mLCs to make favorable contacts with SK and rearrange to form an active complex. This was indeed revealed by the absence of an active site acylation signal in SK2b/mPg and SK1/mPg (data not shown) using the single-turnover substrate, MUGB, in which no turnover was achieved with any chimeric Pg containing the mLC. This failure in the formation of active centers is not influenced by the mHC or hHC. Consequently, it is plausible that sequence variations in the hLC and mLC, particularly the differences in residues 624 to 628, contribute to the lack of activation of mPg by any subform of SK.

In conclusion, we have shown in intact proteins that the binding determinants of hPg for PAM uniquely reside in K2_hPg_. Aside from the interaction between K4_hPg_, K5_hPg_, and N-terminal activation peptide domains of hPg, the K2_hPg_ interaction with LC also strongly modulates the hPg conformation. Whether Pg forms an active complex with SK depends on specific sequences within its LC. The HC dictates the Pg conformation and further influences the overall Pg conformation by interacting with the LC. Thus, once the LC sequence requirement is met, the overall Pg conformation, also affected by ligand binding (e.g., PAM), determines the rate of formation of the active SK–Pg complex, the initial step in the activation of hPg by SK. Most importantly, our results demonstrate the significance of conformational constraints and correct orientation between residues of interacting protein partners in SK-hPg catalysis.

## Experimental procedures

### Construction, expression, and purification of recombinant proteins

*Pg and Pg chimeras.* Parental Pg expression plasmids, hPg-pMT-Puro and mPg-pMT-Puro, were generated by insertion of the cDNAs encoding these proteins into the multiple cloning site of pMT-Puro as described (25, 54). All mutations were constructed from these two parental plasmids. The chimeric Pgs, *viz.,* mHC-hLC (mouse heavy chain–human light chain) and hHC-mLC (human heavy chain–mouse light chain), were generated by overlapping primer-mediated restriction-free cloning (55), with details provided earlier (25). hPg[D^219^N] (human Pg with a [D^219^N] mutation) was constructed with the QuikChange XL site-directed mutagenesis kit (Stratagene). Nucleotide sequencing of each Pg verified the integrity of the constructs. *Drosophila Schneider S2* cells were then transfected with the various Pg/pMT-Puro plasmids. Positive colonies were screened by puromycin resistance, and the Pgs were expressed as previously described (56).

After induction with CuSO_4_, all Pg variants were purified from their culture supernatants using lysine-Sepharose affinity chromatography (37). Protein concentrations of the Pgs were determined by A_280nm_, using the following extinction coefficients (M^-1^cm^-1^): hPg, 152,200; mPg, 162,630; mHC-hLC, 159,650; hHC-mLC, 155,180; and hPg[D^219^N], 152,200. The values were calculated from their amino acid sequence by ExPASy. The purity and homogeneity of the Pgs were assessed by SDS-PAGE using 10% Tris-glycine gels.

*SK.* Detailed descriptions of plasmid construction, expression from *Escherichia coli*, and purification of recombinant SK proteins have been provided in a previous study (15). Briefly, gDNAs encoding *sk1* and *sk2b* were cloned from GAS isolate NS53 (from M. Walker, Queensland, Au) and AP53 (from G. Lindahl, Lund, SE), respectively, using standard methodology. The SKs were cloned into pCR2.1-TOPO (Invitrogen) and sequenced. The *sk1* and *sk2a* genes (minus the signal sequences) were amplified by Phusion Hot Start High-Fidelity DNA Polymerase (New England Biolabs), digested with *Bam*H1 (inserted through the reverse PCR primers), and ligated into *Psh*A1/*Bam*H1-digested pET42a (EMD4Biosciences). The final plasmids contained [glutathione S-transferase-(His)_6_-Factor Xa (FXa) cleavage site-*sk1/sk2b*]. Each plasmid was transformed into BL21/DE3 (New England Biolabs) cells for expression. After induction with isopropyl β-d-1-thiogalactopyranoside, SKs were purified on a Ni+-charged His-bind column, eluted with imidazole, and finally cleaved with FXa (ERL) to yield purified SK with an intact amino-terminus (16).

*PAM.* PAM, without the N-terminal signal peptide and C-terminal LPXTG cell wall anchor region, was cloned by PCR from GAS-AP53 gDNA. A (His)_6_-tag was engineered into the reverse primer for PAM for further facile purification by Ni+-based affinity chromatography. The detailed steps have been described earlier (57).

### SEC-MALS analysis

Molecular weights of Pg variants were determined using an Agilent 1260 Infinity II HPLC system connected to a Wyatt WTC-030S5 (7.6 nm × 300 nm, 5 μm, 300A°) SEC column. The SEC column was coupled to a mini DAWN Treos II light scattering detector and an Optilab T-rex differential refractive index detector (Wyatt Technology Corporation). Pg (100 μl) at a concentration of 1 mg/ml was injected into the SEC column at a flow rate of 0.5 ml/min using PBS, pH 7.4, as the running buffer. Light scattering and refractive index signals of eluted Pg peaks were recorded by respective detectors. The refractive index increment (dη/dc) was set to 0.185 ml/g. The data were analyzed for molar mass determinations using Astra 7.0 software (Wyatt Technology Corporation).

### Analytical ultracentrifugation

Sedimentation velocity experiments were carried out using absorption optics at 20 °C with an initial Pg A_280nm_ of 0.75 (∼0.4 mg/ml) in a Beckman Optima XL-1 analytical ultracentrifuge. The buffer employed was 50 mM sodium phosphate/100 mM NaCl, pH 7.4. Samples were run in three 2-channel centerpieces with sample buffer in the reference channels and Pg in the same buffer in the sample channels. The cells were centrifuged at 30,000 rpm and scans were recorded every 3 min for 12 h. Curves generated from the scans were analyzed for the *S*_*app*_ using Optima XL-A/XL-I software (Beckman coulter). The buffer viscosity and density and the partial specific volume of each Pg were calculated from their compositions using ExPASY. These values were used for correction of the S_app_ to the S_20,w_. Since the protein concentration is so low, the S_20,w_ values are essentially equal to the S^0^_20,w_ values. The effect of EACA on the S_20,w_ of Pg was determined in sample buffers containing 100 mM EACA. The S_20,w_ is reported as mean of three values ± the SD.

For molecular weight determinations, sedimentation equilibrium experiments were conducted at 25 °C. The proteins were diluted to final A_280nm_ of ∼0.1, loaded into six-channel centerpieces, and centrifuged at 20,000 rpm. Scans were recorded hourly until sedimentation equilibrium was attained. Apparent weight average molecular weights for each protein were calculated following data analysis with OptimaXL-A/XL-I software (Beckman Coulter). At least triplicate data were collected for each Pg, and values are reported as mean ± SD.

### Activation of Pg

Pg activation assays were performed in 96-well microtiter plates using the chromogenic substrate, H-D-Val-L-Leu-L-Lys-p-nitroanilide (S2251; Chromogenix), to monitor the continuous generation of Pm at 25 °C through release of p-nitroaniline from S2251. A 200-μl assay mixture in each sample well contained final concentrations of 0.2 μM Pg/0.25 mM S2251/0 or 0.25 μM PAM, in 10 mM Na^+^-Hepes/150 mM NaCl, pH 7.4. The reaction was accelerated by addition of either 5 nM SK2b or 5 nM SK1. Three independent experiments were performed for a given Pg at a specific PAM concentration. The data were analyzed using GraphPad Prism, version 8.0. Initial velocities of activation were calculated as the slope of the linear region of a plot of A_405nm_ against t^2^.

For experiments designed to measure the extent of active site formation in equimolar SK2b–Pg complexes, studies using a fluorometric single turnover substrate MUGB were employed. Pg (5 μM) in 0.1 M sodium phosphate/0.1 M NaCl, pH 6.0, was placed in black 96-well microtiter plates. MUGB was added to a final concentration of 200 μM, and the reaction was accelerated by 10 μM SK2b, with and without 5 μM PAM. Excitation and emission wavelengths were 323 nm and 445 nm, respectively. The reaction progressed until a steady state (∼10 mins) was achieved in the fluorescence intensity of methylumbelliferone generated. Amounts of the methylumbelliferone generated were estimated by extrapolation of the fluorescent steady state curve to zero, thus providing only the presteady state value. The ratio of [methylumbelliferone]/[Pg] present in the well × 100 was taken as the % active site formation in Pg.

A standard curve was constructed by hydrolyzing known concentrations of MUGB with 0.1 M NaOH, followed by adjustment of pH to 6.0 using 1M NaH_2_PO_4_.

### Surface plasmon resonance

Association (k_on_) and dissociation (k_off_) rate constants for the binding of Pg to PAM were determined at 25 °C in a BIAcore X100 (GE Healthcare) system, using HBS-EP^+^ (10 mM Na^+^-Hepes/150 mM NaCl/3 mM EDTA/0.05% polysorbate 20, pH 7.4) as the running buffer for immobilization and binding experiments. PAM (2.5 μg/ml) in 10 mM NaOAc, pH 4.5, was covalently immobilized on a CM-5 chip to a target level of up to 250 response units using the amine coupling kit (BIAcore AB). Prior to immobilization, the surface of the chip was first activated by 0.2 M ethyl-N-dimethylaminopropyl carbodiimide and 0.05 M N-hydrosuccinimide at a flow rate of 5 μl/min. PAM was then injected into flow cell-2 (FC2) to allow immobilization, after which excess nonbound sites on the chip were blocked using 1 M ethanolamine, pH 8.5. Flow cell-1 (FC1) was prepared as a blank cell by omitting the PAM injection. At least three independent binding experiments were conducted for each Pg variant at concentrations ranging from 0 to 50 nM, except for hPg[D^219^N] which was used at concentrations up to 300 nM. Each cycle during binding consists of an association step in which a single concentration of Pg was injected to both FC1 and FC2 at a flow rate of 30 μl/min for 180 s, a dissociation step in which the running buffer was allowed to flow for 300 s, and finally a regeneration step in which 10 mM glycine-HCl, pH 1.5, was injected to prepare the chip surface for the next cycle. Signals from FC1 were subtracted from FC2, and the sensorgrams were best-fit with a 1:1 binding model using BIAevaluation software version 3.0. Dissociation constants (K_D_) were calculated from the average values of k_off_/k_on_. Error values were reported as standard deviations from mean values.

In order to determine the kinetic parameters for the interaction of SK and chimeric Pgs, immobilization of each Pg was achieved by amine coupling on CM-5 chips to target levels of ∼1200 response units as described (15). Binding experiments were conducted at SK1 and SK2b concentrations ranging from 0 to 0.25 μΜ and 0 to 2 μM, respectively, using HBS-EP+/50 μΜ p-nitrophenyl-p’-guanidinobenzoate, which functions similarly to MUGB to maintain the SK–hPg complex as the analyte buffer.

## Data availability

All data are contained within the article.

## Conflict of interest

The authors declare that they have no conflicts of interest with the contents of this article.
