# Supplementary material for: Streptococcus co-opts a conformational lock in human plasminogen to facilitate streptokinase cleavage and bacterial virulence
Source: J Biol Chem. 2020 Nov 24;296:100099. doi: 10.1074/jbc.RA120.016262 (PMC7948469; doi:10.1074/jbc.RA120.016262)
Supplement: Supplementary file 1 — Figure S1 [file mmc1.pdf]

Supplemental Figure 1

A

```
hPg      EPLDDYVNTQGASLFSVTKKQLGAGSIEECAAKCEEDEEFTCRAFQYHSKEQQCVIMAENRKSSIIIRMRDVVLFEKKVYLSECKTGNGKNYRGTMSKTKNGITCQKWSSTSPHRPRFSP 120
mPg      DSLDGYISTQGASLFSLTKKQLAAGGVSDCLAKCEGETDFVCRSFQYHSKEQQCVIMAENSKTSSIIRMRDVILFEKRVYLSECKTGIGNGYRGTMSRTKSGVACQKWGATFPHVPNYSP 120
         : **.*:.********:*****.**.:.:* ****  : :*.**:**************** *:* *******:****:********* *:.******:**.*::****.:* ** *.:**

hPg      ATHPSEGLEENYCRNPDNDPQGPWCYTTDPEKRYDYCDILECEEECMHCSGENYDGKISKTMSGLECQAWDSQSPHAHGYIPSKFPNKNLKKNYCRNPDRELRPWCFTTDPNKRWELCDI 240
mPg      STHPNEGLEENYCRNPDNDEQGPWCYTTDPDKRYDYCNIPECEEECMYCSGEKYEGKISKTMSGLDCQAWDSQSPHAHGYIPAKFPSKNLKMNYCRNPDGEPRPWCFTTDPTKRWEYCDI 240
         :***.************** **********:******:* *******:****:*:***********:****************:***.**** ******* * *********.**** ***

hPg      PRCTTPPPSSGPTYQCLKGTGENYRGNVAVTVSGHTCQHWSAQTPHTHNRTPENFPCKNLDENYCRNPDGKRAPWCHTTNSQVRWEYCKIPSCDSSPVSTEQLAPTAPPELTPVVQDCYH 360
mPg      PRCTTPPPPPSPTYQCLKGRGENYRGTVSVTVSGKTCQRWSEQTPHRHNRTPENFPCKNLEENYCRNPDGETAPWCYTTDSQLRWEYCEIPSCESSASPDQSDSSVPPEEQTPVVQECYQ 360
         ********  .******** ******.*:*****:***:** **** *************:*********: ****:**:**:*****:****:**    :. :  . * * *****:**:

hPg      GDGQSYRGTSSTTTTGKKCQSWSSMTPHRHQKTPENYPNAGLTMNYCRNPDADKGPWCFTTDPSVRWEYCNLKKCSGTEASVVAPPPVVLLPDVETPSEEDCMFGNGKGYRGKRATTVTG 480
mPg      SDGQSYRGTSSTTITGKKCQSWAAMFPHRHSKTPENFPDAGLEMNYCRNPDGDKGPWCYTTDPSVRWEYCNLKRCSETGGSVVELPTVSQEPSGPSDSETDCMYGNGKDYRGKTAVTAAG 480
         .************ ********::* ****.*****:*:*** ********.******:**************:** * .***  * *   *.  : ** ***:****.**** *.*.:*

hPg      TPCQDWAAQEPHRHSIFTPETNPRAGLEKNYCRNPDGDVGGPWCYTTNPRKLYDYCDVPQCAA-PSFDCGKPQVEPKKCPGRVVGGCVAHPHSWPWQVSLRTRF-GMHFCGGTLISPEWV 598
mPg      TPCQGWAAQEPHRHSIFTPQTNPRAGLEKNYCRNPDGDVNGPWCYTTNPRKLYDYCDIPLCASASSFECGKPQVEPKKCPGRVVGGCVANPHSWPWQISLRTRFTGQHFCGGTLIAPEWV 600
         ****.**************:*******************.*****************:* **:   **:*********************:********:****** * ********:****

hPg      LTAAHCLEKSPRPSSYKVILGAHQEVNLEPHVQEIEVSRLFLEPTRKDIALLKLSSPAVITDKVIPACLPSPNYVVADRTECFVTGWGETQGTFGAGLLKEAQLPVIENKVCNRYEFLNG 718
mPg      LTAAHCLEKSSRPEFYKVILGAHEEYIRGSDVQEISVAKLILEPNNRDIALLKLSRPATITDKVIPACLPSPNYMVADRTICYITGWGETQGTFGAGRLKEAQLPVIENKVCNRVEYLNN 720
        ********** **. ********:*     .****.*::*:***..:******** **.***************:***** *::************* **************** *:**.

hPg      RVQSTELCAGHLAGGTDSCQGDSGGPLVCFEKDKYILQGVTSWGLGCARPNKPGVYVRVSRFVTWIEGVMRNN 791
mPg      RVKSTELCAGQLAGGVDSCQGDSGGPLVCFEKDKYILQGVTSWGLGCARPNKPGVYVRVSRFVDWIEREMRNN 793
         **:*******:****.********************************************* ***  ****
```

B

```
hK1      CKTGNGKNYRGTMSKTKNGITCQKWSSTSPHRP-RFSPATHPSEGLEENYCRNPDNDPQGPWCYTTDPEKRYDYCDILEC 79
hK2      CMHCSGENYDGKISKTMSGLECQAWDSQSPHAH-GYIPSKFPNKNLKKNYCRNPDREL-RPWCFTTDPNKRWELCDIPRC 78
hK3      CLKGTGENYRGNVAVTVSGHTCQHWSAQTPHTH-NRTPENFPCKN-DENYCRNPDGKR-APWCHTTNSQVRWEYCKIPSC 77
hK4      CYHGDGQSYRGTSSTTTTGKKCQSWSSMTPHRH-QKTPENYPNAGLTMNYCRNPDADK-GPWCFTTDPSVRWEYCNLKKC 78
hK5      CMFGNGKGYRGKRATTVTGTPCQDWAAQEPHRHSIFTPETNPRAGLEKNYCRNPDGDVGGPWCYTTNPRKLYDYCDVPQC 80

         *    *:.* *. : * .*  ** * :  **      * . *   .   ******* .   ***.**:    :: *.:  *
```

Figure S1. A. Protein sequence alignments of hPg and mPg, showing extensive homology of the two proteins. B. Protein sequence alignments of the five kringle domains of hPg.
